# Influence of Laser Shock Peening on the Bobbin Tool Friction Stir-Welded AW6060 Alloy

**DOI:** 10.3390/ma18020247

**Published:** 2025-01-08

**Authors:** Sebastian Balos, Milan Pecanac, Dragan Rajnovic, Igor Barenyi, Henrieta Chochlikova, Danka Labus Zlatanovic, Jean Pierre Bergmann, Milos Knezev, Slobodan Radisic, Jozef Majerik

**Affiliations:** 1 Faculty of Technical Sciences, University of Novi Sad, 21000 Novi Sad, Serbia; sebab@uns.ac.rs (S.B.); draganr@uns.ac.rs (D.R.); knezev@uns.ac.rs (M.K.); sradisic@uns.ac.rs (S.R.); jozef.majerik@tnuni.sk (J.M.); 2 Faculty of Special Technology, Alexander Dubcek University of Trencin, 911 50 Trencin, Slovakia; igor.barenyi@tnuni.sk (I.B.); henrieta.chochlikova@tnuni.sk (H.C.); 3 Department of Production Technology, Technische Universität Ilmenau, 98693 Ilmenau, Germany; danka.labus-zlatanovic@tu-ilmenau.de (D.L.Z.); jeanpierre.bergmann@tu-ilmenau.de (J.P.B.); 4 Faculty of Economics and Engineering Management, University Business Academy Novi Sad, 21000 Novi Sad, Serbia

**Keywords:** friction stir welding, laser shock peening, microhardness

## Abstract

Friction stir welding (FSW) is a solid-state welding process that uses a rotating tool to soften and stir the base metal, thereby joining it. A special type of tool that has attracted the interest of researchers is the so-called bobbin tool (BTFSW), which, unlike conventional tools with one shoulder, features two shoulders that envelop the base metal from both the top and bottom sides. As a result, significant tensile stresses develop on both sides of the weld, caused by the action of both tool shoulders. In this paper, this issue was addressed by applying laser shock peening (LSP), aiming to introduce compressive stresses, which can be useful as a post-processing technique for BTFSW on both weld sides. It was found that this process completely alters residual stresses in the treated area, from tensile to compressive, through shock waves that impart plastic deformation in the surface layer. It was shown that the LSP effect is more pronounced as the accumulated energy is higher. As a consequence, the microhardness values were significantly increased in the surface and subsurface layers, reaching a maximum depth of 480 to 780 µm for the lowest and highest accumulated laser energy, respectively, while surface roughness increased. While increasing compressive stresses and microhardness in the surface layer is beneficial from the point of view of fatigue resistance, increased roughness has a detrimental effect. Accumulated energy was hereby shown to have a higher effect compared to the maximal energy applied to the specimens.

## 1. Introduction

Friction stir welding (FSW) is a solid-state welding process invented at The Welding Institute (TWI), Cambridge, UK. This process uses a specially designed non-consumable rotational tool, plunged into the base metal and traversed along the weld seam. During this process, the base metal is plastically deformed and intermixed, forming the weld [1,2,3]. By optimising the tool geometry to enhance material flow, and the process parameters, such as the tilt angle, the rotation, and the welding speed, an effective material flow pattern and temperature distribution can be achieved. This optimisation facilitates desirable microstructural evolution and improves the structural integrity of welds [4]. So far, a wide array of materials, including dissimilar materials, have been successfully welded using FSW, providing significant advantages compared to other competing processes. However, FSW has found its niche in joining aluminium alloys, but also in other light alloys [5,6], even steels [7,8] and dissimilar base metals [9,10]. FSW has a number of advantages over other welding processes: a relatively high weld strength, low welding temperatures compared to fusion processes, and, therefore, lower or even non-existent distortion and lower energy consumption. These features make it a “green” welding process, as FSW does not require shielding gas or consumable materials in the form of sticks or wires [11,12]. Furthermore, the base metal is not brought to its melting point, eliminating most of the negative effects associated with cooling and solidification in fusion welding processes, such as hot cracking, segregation, and a flawed microstructure in the fusion zone. However, some concerns remain regarding residual stresses, which differ significantly from those of the base material. It is widely accepted that residual stresses can have a substantial impact on the performance of components, including FSW joints [13]. Premature failure and distortion may be the result of excessive tensile stresses, as shown in several studies [14,15,16]. Thus, there is a strong tendency towards increasing tensile stresses, as the result of the rotational and longitudinal movement of the FSW tool, stirring the base material.

Residual stresses depend upon the weld zone and parameters, mainly rotation speed. Lemos et al. [17] demonstrated that high rotational speeds (1000, 1200 rpm) led to higher tensile residual stresses and microhardness compared to a low rotational speed (200 rpm). Furthermore, at 1000 and 1200 rpm, there is a slightly larger difference in residual stresses between the advancing side (AS) and the retreating sides (RS) compared to the weld obtained at low-rpm settings. This indicates that welds obtained at a lower rotational speed result in a more uniform material flow. Hadi et al. [18], in their study of FSW of dissimilar aluminium alloys AW2024 and AW7075, showed that residual stresses varied across different weld zones depending on the arrangement of the base materials, specifically whether the AS and RS were oriented towards the AW2024 or AW7075 base material. An increase in tool rotation speed also widens the regions of increased residual stresses within the heat-affected zone (HAZ) and the nugget zone (NZ). Furthermore, when AW2024 is positioned on the AS, the maximum tensile residual stress occurs in AW7075 on the RS, while, if AA7075 is on the AS, the residual stress peaks on the AS and RS are similar in magnitude. Clearly, tensile residual stresses have a significant effect on the performance of the weld, and they need to be reduced in order to increase the integrity of the structure, particularly under fatigue conditions [19].

One of the most efficient ways of reducing tensile residual stresses and converting them into more convenient compressive stresses is the application of laser shock peening (LSP) [20,21]. In LSP, the surface of the material is irradiated with repeated laser pulses. The vaporised material close to the surface causes the intensive ionisation of atoms, generating rapidly expanding plasma [22]. This, in turn, creates a pressure pulse acting on the surface, introducing mechanical shock waves. These waves cause localised plastic deformation in the surface layer of the treated material, which subsequently induces beneficial compressive residual stresses [23]. To enhance the plasma effect, a confinement is applied, usually in the form of a water layer [23,24,25] or a solid, in the form of glass [26,27] or a polymer [28,29]. Besides the confinement, there are several other important parameters of the LSP process, such as the pulse wavelength, duration, shape, and energy [30]. The influence of LSP is not limited to achieving a higher fatigue performance, but also corrosion resistance, wear, and even tensile strength of the treated specimens [31,32].

In this work, an effort was made to combine two technologies, FSW and LSP, with water confinement in order to reduce surface tensile residual stresses. A special type of bobbin tool FSW (BTFSW) was used, which uses a two-sided tool, enveloping the base metal. This approach creates two flat, formed surfaces at the both the surface and root of the weld, eliminating the unwelded root—a common source of defects, which often compromises mechanical properties, particularly bending and fatigue properties. Furthermore, bobbin tool design eliminates the need for a backing plate, simplifying the process and making it more suitable for industrial use, as tool–base metal interference is fixed by tool geometry [33,34].

## 2. Materials and Methods

**Figure 1:**
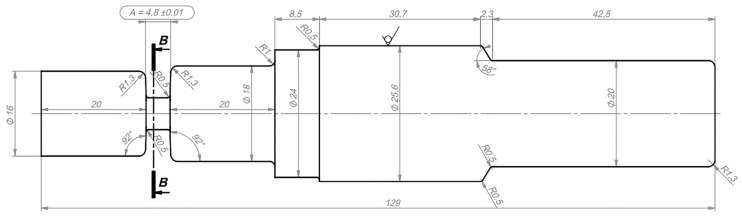
Tool geometry used in BTFSW.

The base material (BM) used in this study was AW6060 T66. The chemical composition, tested by the G.N.R. MiniLab 150 optical emission spectrometer (OES) device (Milan, Italy), is shown in Table 1. The BTFSW process was performed on 200 × 60 mm specimens, 5 mm thick, using the Deckel Maho FP3-50 CNC milling machine (Katzwinkel, Germany) and AISI H13 hot work tool steel hardened to 53 HRC, as shown in Figure 1. Four specimens were welded with a constant welding speed of 20 mm/min and a tool rotation speed of 1120 rpm.

The LSP process was applied to three specimens, using the Quantel Twins Nd:YAG 10 ns pulsed laser (Paris, France). The wavelength used was 532 nm, the spot size Ø1 mm, and ½ overlapping pattern was applied to form the treated area of 15 × 5 mm on the face and 13 × 5 mm on the root side, which corresponds to the flat formed weld surfaces. The laser treatment was carried out in distilled water confinement. LSP was conducted at 0.235 J in one and three passes (designated 0.235 × 1 and 0.235 × 3) and at 0.47 J energy in one and three passes (0.47 × 1 and 0.47 × 3), Table 2.

**Figure 2:**
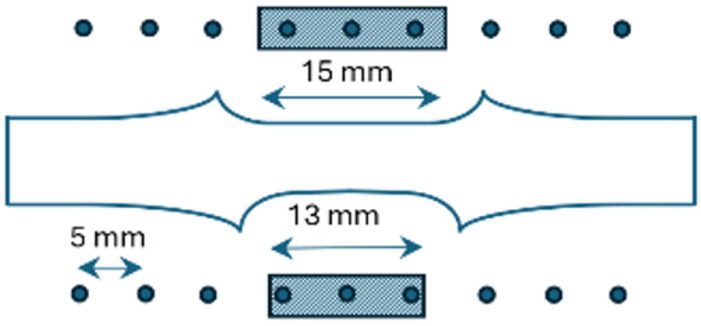
Residual stress measurement pattern in the welded specimen; in the centre is the depiction of the common BTFSW profile.

A metallographic examination was carried out using the Struers preparation laboratory, which included cutting, mounting, grinding (SiC abrasive papers, grit sizes from P100 to P2500), and polishing (diamond suspensions of 6, 3, 1, and ¼ µm). Electrolytic etching was performed at 35 V for 30 s, using Barker’s etchant (200 mL HBF_4_ + 800 mL H_2_O). To examine the prepared specimens, a Leitz Orthoplan light microscope (Wetzlar, Germany) (LM) was used. Microhardness was tested by the Wilson Tukon 1102 device (Lake Bluff, IL, USA), with the application of a 50 g load. Measurements were taken in the LSP zone, starting from the surface towards the centre of the specimen, in both laser-treated and untreated specimens, through the stir zone, to a depth of 1000 µm. Indentations were spaced at intervals of 100 µm. Residual stresses were measured using the X-ray diffraction method on the surface specimen. Measurements points were spaced 5 mm apart, beginning at the centreline of the weld, towards the base metal, as shown in Figure 2. Three central measurement spots were affected by LSP. Measurements were performed on each of three welded specimens, before and after LSP, using the Rigaku MiniFlex 600 device (Tokyo, Japan), with Cr-Kα tube, (311) diffracted plane, 139.3° Bragg angle of diffraction (2θ), 15 mA current, and 40 kV voltage. Longitudinal stresses, as representative and much higher compared to transverse stresses, were reported. Also, surface roughness was measured by SJ-210 (Mitutoyo, Kawasaki, Japan), before and after LSP treatment.

## 3. Results and Discussion

**Figure 3:**
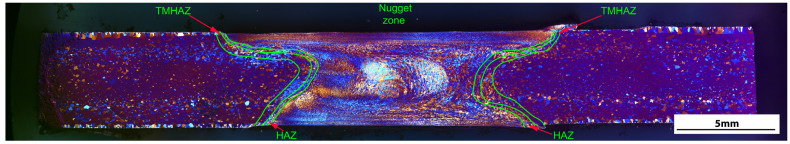
Macro image of the BTFSW specimen.

A macro image of the BTFSW specimen is shown in Figure 3. The hourglass shape of the weld is similar to other researchers’ results where a bobbin-type FSW tool was used [35]. This characteristic shape results from the tool rotation and the interaction between the base metal (BM) and two shoulders of the bobbin tool.

**Figure 4:**
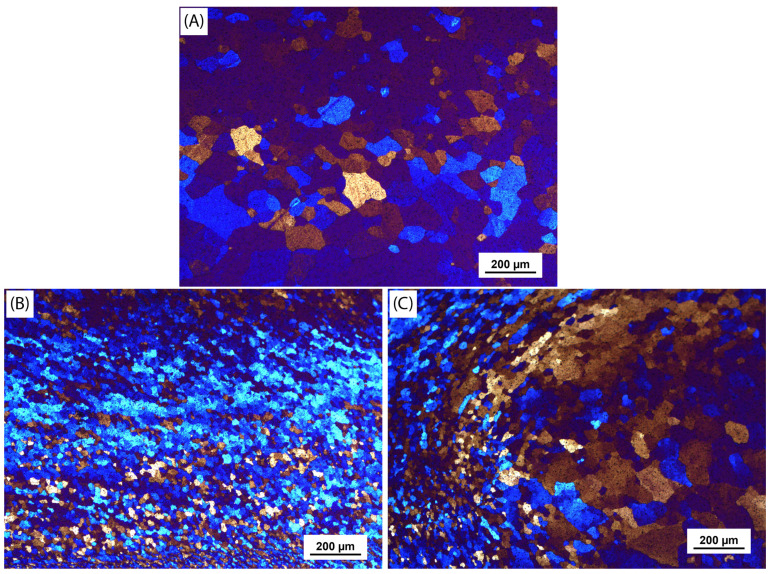
Microstructures of the (**A**) base material, (**B**) stir zone, and (**C**) transition zone showing TMAZ (left) and HAZ (right).

The central stir zone (SZ) is formed by the tool pin, which interconnects the shoulders. It encompasses all zones that are common for the conventional FSW: SZ, the thermomechanical-affected zone (TMAZ), and the heat-affected zone (HAZ), as well as BM. The corresponding microstructures are given in Figure 4.

As in conventional FSW, the microstructures of the SZ and HAZ exhibit uniaxial grains. However, in the HAZ, the grains are approximately of a similar size to those in the BM, due to the heat imparted by the tool and recrystallisation, unlike those in the SZ. In SZ, the tool induces forces that cause intense plastic deformation, which breaks up the existing grain structure and promotes the nucleation of new, smaller grains, within the process of dynamic recrystallisation. In other words, as the material flows around the rotating tool, the high strain rate and shear forces cause rapid grain rotation, stretching, and fragmentation, which lead to the formation of smaller, finer grains in the stir zone.

The TMAZ, located between the SZ and HAZ, is influenced by both thermal and mechanical effects from the tool. The material undergoes plastic deformation and is subjected to temperatures that are significant but not as extreme as in the SZ.

**Figure 5:**
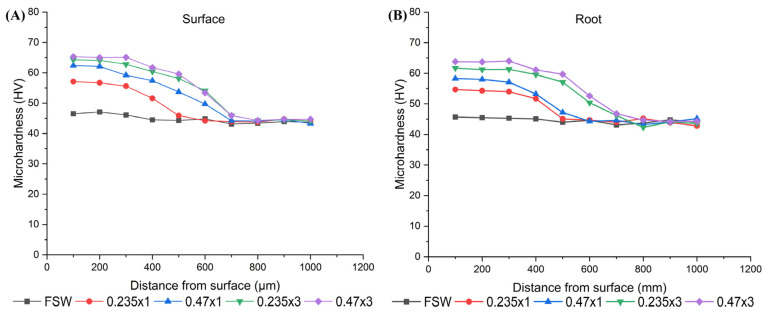
Microhardness depths in relation to specimen treatment. (**A**) Surface (**B**) Root.

The microhardness results are shown in Figure 5. The average microhardness of welds created by BTFSW without LSP treatment was measured at 45 HV both at the weld face and root. However, specimens treated with LSP showed a considerable rise in microhardness, particularly near the surface of the weld, both in the weld face and root, where similar trends were observed.

The results show that the microhardness values gradually decrease with increasing depth from the surface. Among the specimens, the highest increase in microhardness was obtained in specimen 0.47 × 3 with the highest energy input, followed by specimens 0.235 × 3, 0.47 × 1 and 0.235 × 1 with the lowest accumulated energy.

For specimens 0.47 × 3 and 0.235 × 3, the baseline microhardness of 45 HV0.3 (average SZ values for the no-LSP specimen) was reached at the depth of 700 µm at the weld face and root, respectively.

For specimens 0.47 × 1, the baseline value was reached at 600 µm at the face and 500 µm at the root, while for specimen 0.235 × 1 it was reached at 500 µm for both the face and the root. These depths can be considered the effective LSP hardening depths for BTFSW specimens. As no change in grain size was noted before and after the LSP process, it is supposed that dislocation hardening plays a major role, causing interactions between dislocations and dislocations with other phases within the material, as explained in [36].

**Figure 6:**
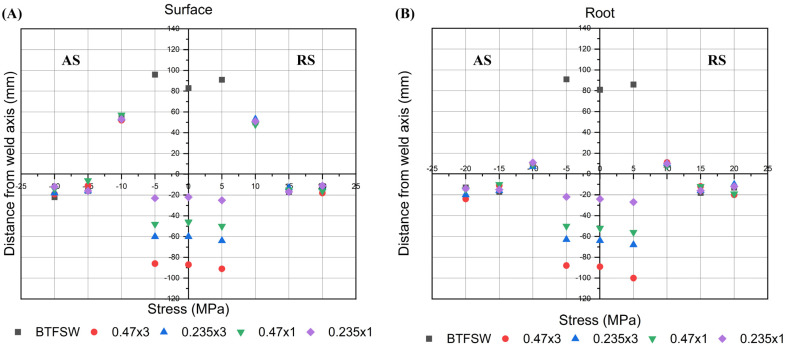
Residual stresses of FSW and LSP-treated specimens: (**A**) surface and (**B**) root of the weld.

The results of residual stress measurements at the surface, the most critical part of the material, are presented in Figure 6. The base material exhibited mildly compressive residual stresses on both sides of the welded specimen, at 15 and 20 mm from the centreline. At 10 mm from the centreline, mild tensile stresses were observed on the root side, while considerable tensile stresses were obtained on the surface side. Those surface stresses resulted from the deformation caused by the tool’s sinking into the base material and the pulling action induced by tool rotation.

High tensile stresses were measured on both the surface and root sides of the area that was in close contact and under the influence of the tool shoulders. These values were higher at 5 mm from the centreline than in the centre, with the maximum tensile stresses were obtained on the advancing side (AS) of the BTFSW specimen. This stress peak occurred on the advancing side, since the tool welding speed was added to the circumferential speed, similar to the results obtained in [37]. However, the differences in stress values between the +5 mm and −5 mm sides were relatively small, probably due to the relatively low welding speed used.

The same trend was evident in both the surface and root areas and could be attributed to the specific geometry of the bobbin tool, which has two shoulders connected by a pin. This is in contrast to the conventional tool, which produces lower tensile stresses in the weld root in a much more pronounced “M”-type pattern [38].

The effect of LSP is beneficial in converting tensile residual stresses into compressive ones. Longitudinal stresses were reported as being representative to show the effect of LSP post treatment. The results depicted in Figure 6 clearly indicate that, in the region treated with LSP (±5 mm from the 0 point), compressive stresses were observed, reaching −100 MPa at the weld root on the retreating side (RS). Furthermore, it can be seen that a higher cumulative energy resulted in higher compressive stress, regardless of the laser energy applied during the LSP process. Therefore, it can be concluded that the residual stresses in the central section followed the same trend as the microhardness results in the surface region, as shown in Figure 5.

The results of the surface roughness measurements of the specimens are shown in Table 3. The results indicate that LSP treatment leads to increases in the average (Ra) and maximum roughness (Rz), compared to the values measured before LSP. Additionally, it can be seen that a higher accumulated laser treatment energy results in a more significant increase in the roughness parameters. This increase in roughness after LSP treatment can be attributed to the increased intensity of plastic deformation during LSP, which can also explain the higher increase in roughness parameters in specimens treated with a higher accumulated energy. These results are consistent with those obtained for the LSP of machined surfaces of AA7050-T7451 by Attolico et al. [39], as well as Ti-6Al-4V [40]. This is in a strong contrast to the effect of LSP of 3D-printed specimens, that is, selective laser melting (SLM) specimens, where the surface roughness parameters suggest the smoothing of the surface due to the effect of deceasing the sharpness of the deep valleys and partially remelting the loosely bonded particles on the peaks [40]. An increased surface roughness has a detrimental effect on fatigue resistance [37]; however, compressive stress and increased surface microhardness prove beneficial for the same purpose, as well as corrosion resistance [38,39].

## 4. Conclusions

The results obtained within the framework of this research, while acknowledging its limitations, led to the following conclusions:

- Residual stresses in the surface, the most critical region of the untreated base material, were tensile in nature, due to the pulling action of the tool on both sides of the weld.
- The highest compressive stresses in the welded specimens were found on the advancing side within the surface of the weld, due to the most intensive pulling action of the tool shoulder.
- Laser shock peening had a significant effect on converting tensile stresses into compressive stresses.
- The accumulated laser energy played a dominant role over the laser shock peening process energy, significantly influencing the increase in induced compressive stresses. As the accumulated energy was higher, the induction of compressive stresses was more pronounced.
- Surface roughness increased as a result of the laser shock peening process. The results of average and maximum roughness obtained closely followed the accumulated energy, where a higher accumulated energy led to a higher increase in roughness.

## Figures and Tables

**Table 1:** Chemical composition of the base material [mass. %].

	Cr	Cu	Fe	Mg	Mn	Si	Ti	Zn	Al
BM	0.013	0.043	0.205	0.42	0.055	0.532	0.016	0.026	balance

**Table 2:** LSP parameters.

Specimen Designation	Laser Energy [J]	No. of Passes	Accumulated Energy [J]
0—control sample	0	0	0
0.235 × 1	0.235	1	0.235
0.235 × 3	0.235	3	0.705
0.47 × 1	0.47	1	0.47
0.47 × 3	0.47	3	1.41

**Table 3:** Average roughness (Ra) and maximum roughness (Rz) before and after LSP treatment.

	0.235 × 1		0.47 × 1		0.235 × 3		0.47 × 3	
	Before	After	Before	After	Before	After	Before	After
Ra [µm]	1.598	2.003	1.537	2.034	1.587	2.192	1.596	2.367
Rz [µm]	6.669	8.039	6.672	8.266	6.383	8.682	6.276	8.835

## Data Availability

The original contributions presented in this study are included in the article. Further inquiries can be directed to the corresponding author.

## References

[1] Shrivastava A., Krones M., Pfefferkorn F.E. (2015). Comparison of Energy Consumption and Environmental Impact of Friction Stir Welding and Gas Metal Arc Welding for Aluminum. CIRP J. Manuf. Sci. Technol..

[2] Balos S., Sidjanin L. (2014). Effect of Tunneling Defects on the Joint Strength Efficiency Obtained with FSW. Mater. Technol..

[3] Labus Zlatanović D., Bergmann J.P., Baloš S., Janjatović P., Rajnović D., Šidjanin L. (2023). Influence of Strain Rate on Metallurgical and Mechanical Properties of Friction Stir Spot Welded Aluminium Joints. Adv. Technol. Mater..

[4] Singh Sidhu M., Chatha S.S. (2012). International Journal of Emerging Technology and Advanced Engineering Friction Stir Welding-Process and Its Variables: A Review. Certif. J..

[5] Singh K., Singh G., Singh H. (2018). Review on Friction Stir Welding of Magnesium Alloys. J. Magnes. Alloy..

[6] Desai A.M., Khatri B.C., Patel V., Rana H. (2020). Friction Stir Welding of AZ31 Magnesium Alloy: A Review. Mater. Today Proc..

[7] Mohan D.G., Wu C.S. (2021). A Review on Friction Stir Welding of Steels. Chin. J. Mech. Eng. (Eng. Ed.).

[8] Liu F.C., Hovanski Y., Miles M.P., Sorensen C.D., Nelson T.W. (2018). A Review of Friction Stir Welding of Steels: Tool, Material Flow, Microstructure, and Properties. J. Mater. Sci. Technol..

[9] Mishra A. (2019). Friction Stir Welding of Dissimilar Metal Joints. Materwiss. Werksttech..

[10] Stephen Leon J., Bharathiraja G., Jayakumar V. (2020). A Review on Friction Stir Welding in Aluminium Alloys. Proceedings of the IOP Conference Series: Materials Science and Engineering.

[11] Defalco J. (2006). Friction Stir Welding vs. Fusion Welding. Weld. J..

[12] Balos S., Sidjanin L., Dramicanin M., Zlatanovic D.L., Antic A. (2016). FSW Welding of Al-Mg Alloy Plates with Increased Edge Roughness Using Square Pin Tools of Various Shoulder Geometries. Mater. Tehnol..

[13] Kumar N., Mishra R.S., Baumann J.A. (2014). Residual Stresses in Friction Stir Welding.

[14] Brewer L.N., Bennett M.S., Baker B.W., Payzant E.A., Sochalski-Kolbus L.M. (2015). Characterization of Residual Stress as a Function of Friction Stir Welding Parameters in Oxide Dispersion Strengthened (ODS) Steel MA956. Mater. Sci. Eng. A.

[15] Richter-Trummer V., Suzano E., Beltrão M., Roos A., dos Santos J.F., de Castro P.M.S.T. (2012). Influence of the FSW Clamping Force on the Final Distortion and Residual Stress Field. Mater. Sci. Eng. A.

[16] Steuwer A., Barnes S.J., Altenkirch J., Johnson R., Withers P.J. (2012). Friction Stir Welding of HSLA-65 Steel: Part II. The Influence of Weld Speed and Tool Material on the Residual Stress Distribution and Tool Wear. Metall. Mater. Trans. A Phys. Metall. Mater. Sci..

[17] Lemos G.V.B., Farina A.B., Nunes R.M., Da Cunha P.H.C.P., Bergmann L., Dos Santos J.F., Reguly A. (2019). Residual Stress Characterization in Friction Stir Welds of Alloy 625. J. Mater. Res. Technol..

[18] Hadji I., Badji R., Gaceb M., Kherrouba N., Rabahi L. (2019). Investigation of the Effect of Aluminum Alloy Position on Residual Stresses in Dissimilar Fsw Weld by Using the Ultrasonic Method. Proceedings of the IOP Conference Series: Materials Science and Engineering.

[19] McClung R.C. (2007). A Literature Survey on the Stability and Significance of Residual Stresses during Fatigue. Fatigue Fract. Eng. Mater. Struct..

[20] Kalentics N., Boillat E., Peyre P., Ćirić-Kostić S., Bogojević N., Logé R.E. (2018). Tailoring Residual Stress Profile of Selective Laser Melted Parts by Laser Shock Peening. Addit. Manuf..

[21] Kalentics N., de Seijas M.O.V., Griffiths S., Leinenbach C., Logé R.E. (2020). 3D Laser Shock Peening–A New Method for Improving Fatigue Properties of Selective Laser Melted Parts. Addit. Manuf..

[22] Peyre P., Fabbro R. (1995). Laser Shock Processing: A Review of the Physics and Applications. Opt. Quantum Electron..

[23] Ding K., Ye L. (2006). Laser Shock Peening Performance and Process Simulation.

[24] Martí-López L., Ocaña R., Piñeiro E., Asensio A. (2011). Laser Peening Induced Shock Waves and Cavitation Bubbles in Water Studied by Optical Schlieren Visualization. Phys. Procedia.

[25] Polese C., Glaser D., Polese C., Glaser D., Bedekar R. Water Confinement Influences on the Laser Shock Peening Process Water Confinement Influences on the Laser Shock Peening Process. Proceedings of the 30th International Congress on High-Speed Imaging & Photonics.

[26] Sandmann P., Keller S., Kashaev N., Ghouse S., Hooper P.A., Klusemann B., Davies C.M. (2022). Influence of Laser Shock Peening on the Residual Stresses in Additively Manufactured 316L by Laser Powder Bed Fusion: A Combined Experimental–Numerical Study. Addit. Manuf..

[27] Wu X., Duan Z., Song H., Wei Y., Wang X., Huang C. (2011). Shock Pressure Induced by Glass-Confined Laser Shock Peening: Experiments, Modeling and Simulation. J. Appl. Phys..

[28] Li Y., Ren Z., Jia X., Yang W., Nassreddin N., Dong Y., Ye C., Fortunato A., Zhao X. (2021). The Effects of the Confining Medium and Protective Layer during Femtosecond Laser Shock Peening. Manuf. Lett..

[29] Le Bras C., Rondepierre A., Seddik R., Scius-Bertrand M., Rouchausse Y., Videau L., Fayolle B., Gervais M., Morin L., Valadon S. (2019). Laser Shock Peening: Toward the Tse of Pliable Polid Polymers for Confinement. Metals.

[30] Sticchi M., Staron P., Sano Y., Meixer M., Klaus M., Rebelo-Kornmeier J., Huber N., Kashaev N. (2015). A Parametric Study of Laser Spot Size and Coverage on the Laser Shock Peening Induced Residual Stress in Thin Aluminium Samples. J. Eng..

[31] Cao X., Wu J., Zhong G., Wu J., Chen X. (2024). Laser Shock Peening: Fundamentals and Mechanisms of Metallic Material Wear Resistance Improvement. Materials.

[32] Jing Y., Fang X., Xi N., Chang T., Duan Y., Huang K. (2023). Improved Tensile Strength and Fatigue Properties of Wire-Arc Additively Manufactured 2319 Aluminum Alloy by Surface Laser Shock Peening. Mater. Sci. Eng. A.

[33] Pecanac M., Labus Zlatanovic D., Kulundzic N., Dramicanin M., Lanc Z., Hadzistevic M., Radisic S., Balos S. (2022). Influence of Tool and Welding Parameters on the Risk of Wormhole Defect in Aluminum Magnesium Alloy Welded by Bobbin Tool FSW. Metals.

[34] Balos S., Labus Zlatanovic D., Kulundzic N., Janjatovic P., Dramicanin M., Lanc Z., Hadzistevic M., Radisic S., Rajnovic D., Pecanac M. (2023). Influence of Tool–Base Metal Interference on the Performance of an Aluminium–Magnesium Alloy Joined via Bobbin Tool Friction Stir Welding. Metals.

[35] Kamble L.V., Soman S.N. (2019). Influence of Bobbin Tool Design on Quality of Welds Made by FSW of Aluminium Alloys. Mater. Today Proc..

[36] Oh M.C., Yeom H., Jeon Y., Ahn B. (2015). Microstructural Characterization of Laser Heat Treated Aisi 4140 Steel with Improved Fatigue Behavior. Arch. Metall. Mater..

[37] Li T., Shi Q.Y., Li H.K., Wang W., Cai Z.P. (2008). Residual Stresses of Friction Stir Welded 2024-T4 Joints. Mater. Sci. Forum.

[38] Li T., Shi Q., Li H., Wang W. (2002). Residual Stresses for Friction Stir Welded Al Sheets. Hanjie Xuebao/Trans. China Weld. Inst..

[39] Attolico M.A., Barile C., Casavola C., Moramarco V., Furfari D., Busse D.O. (2022). Effects of Laser Shock Peening on Surface Roughness and Residual Stress of AA 7050-T7451. J. Mater. Eng. Perform..

[40] Dyer K., Ghadar S., Zulić S., Rostohar D., Asadi E., Molaei R. (2024). The Effect of Laser Shock Peening (LSP) on the Surface Roughness and Fatigue Behavior of Additively Manufactured Ti-6Al-4V Alloy. Coatings.

